# Maternal and Neonatal Immune Responses Following COVID-19 Infection and Vaccinations in Pregnancy

**DOI:** 10.3390/vaccines10122019

**Published:** 2022-11-26

**Authors:** Shlomi Toussia-Cohen, Omer Nir, Ravit Peretz-Machluf, Shiran Bookstein-Peretz, Omri Segal, Keren Asraf, Ram Doolman, Gili Regev-Yochay, Yoav Yinon

**Affiliations:** 1 The Department of Obstetrics and Gynecology, Chaim Sheba Medical Center, Tel-Hashomer, Ramat-Gan 52621, Israel; 2 The Sackler Faculty of Medicine, Tel-Aviv University, Tel-Aviv 69978, Israel; 3 The Dworman Automated-Mega Laboratory, Chaim Sheba Medical Center, Tel-Hashomer, Ramat-Gan 52621, Israel; 4 Infection Prevention & Control Unit, Chaim Sheba Medical Center, Tel-Hashomer, Ramat-Gan 52621, Israel

**Keywords:** adverse effects, BNT162b2 vaccine, COVID-19, pregnancy, SARS-CoV-2 IgG serum levels, third (booster) dose

## Abstract

The objective of the study was to compare the maternal and neonatal humoral immune responses among different groups of women, namely those vaccinated by the BNT162b2 vaccine, not vaccinated, and COVID-19-recovered parturient women at the time of delivery. This is a prospective cohort study of pregnant women, divided into four groups: Group A “Recovered”—recovered and not vaccinated. Group B “Second Vaccination”—first and second doses only. Group C “Third Vaccination”—third dose. Group D “No Third Vaccination”—women eligible for the third dose of the vaccine but did not receive it. Maternal and umbilical cord blood were sampled and tested for SARS-CoV-2 IgG antibodies on admittance to labor and immediately postpartum, respectively. Maternal serum SARS-CoV-2 IgG levels were significantly higher among Group C compared to Group B (741.6 (514.5–1069) vs. 333.5 (327–340.2), respectively). Both groups had higher antibody levels compared to Groups A and D (113.5 (61.62–209.1) and 57.99 (32.93–102.1), respectively). Similarly, umbilical cord blood SARS-CoV-2 IgG levels were also highest among Group C compared to the other three groups (1269 (953.4–1690) vs. Group B, 322.6 (305.6–340.5), Group A, 109 (49.01–242.6), and Group D, 103.9 (48.59–222), respectively). In conclusion, pregnant women who were fully vaccinated with three dosages before delivery generated the highest levels of maternal and neonatal SARS-CoV-2 IgG antibodies.

## 1. Introduction

The coronavirus disease 2019 (COVID-19) pandemic has led to substantial morbidity and mortality globally [1]. Moreover, COVID-19 in pregnant women poses increased risk for severe maternal outcomes compared to nonpregnant women, including the need for invasive ventilation and admission to an intensive care unit (ICU) and even death [2,3,4].

Following reports regarding the maternal morbidity associated with COVID-19 in pregnancy, the Israeli Ministry of Health decided to vaccinate pregnant women with the BNT162b2 vaccine. Studies have shown encouraging results regarding the safety of the first two doses of the vaccine with respect to maternal, obstetric, and neonatal outcomes, along with encouraging effectiveness including the ability to generate a sufficient humoral immune response among pregnant women, resulting in a transfer of SARS-CoV-2 IgG to their fetuses [5,6,7,8,9,10,11,12,13].

The outbreak of the B.1.617.2 (delta) variant on July 2021, along with data suggesting reduced efficacy of the BNT162b2 vaccine over time, led to a decision of the Israeli Ministry of Health to vaccinate people older than 60 years, who had received the second dose at least 5 months before, with a third (booster) dose of the BNT162b2 vaccine. The booster dose resulted in decreased rates of infection, as well as a decrease in COVID-19-related severe illness and death, for the vaccinated population [14,15,16,17]. Following this positive data, the Israeli Ministry of Health issued a recommendation and began to vaccinate high-risk sectors of the population including pregnant women with the third dose of the vaccine. Recent studies have shown that, similar to the first and second doses of the BNT162b2 vaccine, the third dose is also safe in pregnancy and effective in generating a significant humoral immune response of SARS-CoV-2 IgG in pregnant women and their fetuses. [18,19,20,21].

The objective of this study was to compare the maternal and neonatal humoral immune responses among different groups of vaccinated, nonvaccinated, and COVID-19-recovered parturient women at the time of delivery.

## 2. Materials and Methods

This was a prospective cohort study of pregnant women in a single tertiary medical center conducted at two different time frames.

First Time Frame

The first time frame was between February and March 2021 and included pregnant women vaccinated with the first two doses of the BNT162b2 (Pfizer/BioNTech) vaccine during pregnancy and COVID-19-recovered pregnant women upon admittance for delivery. In this time frame, vaccinated women were defined as pregnant women who were vaccinated by 2 dosages of the BNT162b2 vaccine at least two weeks before admittance for delivery. COVID-19-recovered women were defined as pregnant women with documented positive polymerase chain reaction (PCR) tests for SARS-CoV-2 at least 10 days prior admittance for delivery and who were asymptomatic for at least 3 days. These women did not receive any vaccination at all.

Second Time Frame

The second time frame was between August and November 2021 (prior to the outbreak of the Omicron variant) and included pregnant women who were vaccinated by a third (booster) dose of the BNT162b2 vaccine and pregnant women who had received the first and second doses of the vaccine only, were eligible for the third dose of the vaccine, but chose not receive it. In this time frame, all women had received two doses of BNT162b2 at least 5 months prior to their inclusion. Women with a positive PCR for SARS-CoV-2 before or during the study period were excluded.

Study Groups

**Figure 1:**
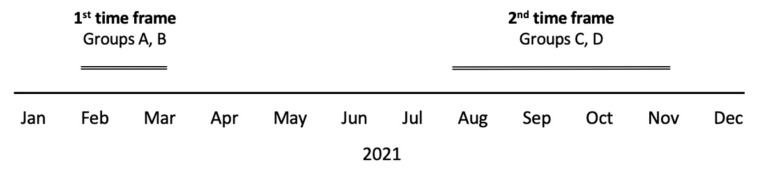
Study Flow Timeline Diagram. Group A “Recovered”; Group B “Second Vaccination”; Group C “Third Vaccination”; Group D “No Third Vaccination”.

Study flow timeline diagram is presented in Figure 1. In the first time frame, Group A “Recovered” were women previously infected and not vaccinated. Group B “Second Vaccination” were women vaccinated by the first and second doses only. In the second time frame, Group C “Third Vaccination” were women vaccinated by the third dose, and Group D “No Third Vaccination” were women eligible for the third dose (received second dose at least 5 months earlier) but did not receive it.

Data Collection

Data regarding medical and demographic background, SARS-CoV-2 infection history, and obstetric complications were collected upon admittance to the labor room.

Hypertensive Disorders of Pregnancy (HDP) were defined as the presence of gestational hypertension or preeclampsia according to the ACOG’s most recent practice bulletin [22]. Gestational diabetes mellitus (GDM) was defined according to the values proposed by Carpenter and Coustan [23]. Smoking was defined as smoking at least one cigarette (or equivalent) per day.

Blood Sample Collection

Maternal and umbilical cord blood samples were collected on admittance to the labor room and immediately postpartum, respectively. Samples were centrifuged at 4000× *g* for 4 min at room temperature. Serum was tested for IgG antibodies against the SARS-CoV-2 spike RBD using the commercial automatic chemiluminescent microparticle immunoassay (CMIA) SARS-CoV-2 IgG II Quant (Abbott, IL, USA) according to manufacturer’s instructions.

Statistical Analysis

Data are described using mean ± standard deviation (SD). To measure the antibody-mediated immune response, the observed geometric mean titer (GMT) and its 95% confidence interval were calculated. Univariate analyses were performed by t-test for normally distributed continuous variables. Chi-square test or Fisher’s exact test were used for categorical variables. A one-way ANOVA test was performed to compare maternal and neonatal blood serology for SARS-CoV-2-specific antibodies and to compare gestational age at the last dose of vaccination and time between vaccination/infection to delivery among the 4 groups. In all comparisons, one-way ANOVA revealed a statistically significant difference between at least two groups. Because equal variances were not assumed in all comparisons, A Dunnett’s T3 test was performed for post hoc multiple comparisons. Significance was accepted at *p* ≤ 0.05. All analyses were conducted using SPSS 25 (SPSS Inc., Chicago, IL, USA).

## 3. Results

### Description of the Experimental Results

One-hundred and thirteen pregnant women were included in the study overall and divided into four different groups: **Group A “Recovered”** included 10 pregnant women with previously documented PCR tests for SARS-CoV-2 who were not vaccinated at all. **Group B “Second Vaccination”** included 57 pregnant women who were vaccinated by the first and second doses only. **Group C “Third Vaccination”** included 38 pregnant women who were vaccinated by the third (booster) dose. **Group D “No Third Vaccination”** included eight pregnant women who were eligible for the third (booster) dose of the vaccine but did not receive it.

Baseline maternal and obstetric characteristics are presented in Table 1. There were no significant differences among the groups in all the collected parameters except for a significant difference in the rates of pregestational diabetes between Group A (2 women) and all the other groups (1 woman in Group B, 0 women in Group C, and 0 women in Group D, *p* = 0.003).

Maternal and neonatal blood serology for SARS-CoV-2-specific antibodies for the different groups are presented in Table 2. Maternal serum SARS-CoV-2 IgG levels were significantly higher among women who received the third dose of the vaccine (Group C) compared to women who received only the first and second doses of the vaccine (Group B) (741.6 (514.5–1069) vs. 333.5 (327–340.2), respectively). Both groups had higher maternal serum SARS-CoV-2 IgG levels compared to Groups A and D (113.5 (61.62–209.1) and 57.99 (32.93–102.1), respectively). Similarly, umbilical cord blood SARS-CoV-2 IgG levels were also highest among Group C women compared to the other three groups (1269 (953.4–1690) vs. Group B, 322.6 (305.6–340.5), Group A, 109 (49.01–242.6), and Group D, 103.9 (48.59–222), respectively), and SARS-CoV-2 IgG levels of Group B umbilical cord blood were significantly higher compared to umbilical cord blood IgG levels of Groups A and D.

Of note, Group C women were vaccinated earlier in gestation compared to Group B (23.81 ± 6.84 vs. 36.35 ± 1.86 weeks, respectively, *p* < 0.001), and therefore the time interval between vaccination and delivery was significantly longer in this group compared to Group B (106.95 ± 50.10 vs. 29.67 ± 55.99 days, respectively, *p* < 0.001). (Table 3).

## 4. Discussion

In this study, we compared the maternal and neonatal humoral immune responses of the Pfizer/BioNTech BNT162b2 vaccine among different groups of vaccinated, nonvaccinated, and COVID-19-recovered parturient women at the time of delivery.

In terms of maternal serology, we observed the highest levels among women vaccinated during pregnancy with the third dose and the lowest levels among recovered women and women who received their second dose months before and were not vaccinated with the third dose. Correlating with the maternal serology results, infants of women vaccinated by the third dose had the highest SARS CoV-2 IgG umbilical cord blood levels compared to all other three groups.

These data suggest that the best maternal humoral response is achieved following the vaccine’s third dose, and due to the transfer of vaccine-induced IgG antibodies across the placenta, their infants achieved the best humoral immunity as well.

Of note, there was a longer time interval between vaccination and delivery in the third-dose group compared to the first- and second-dose groups, and higher maternal and fetal serum SARS CoV-2 IgG levels were observed at delivery. This finding supports the efficacy and importance of the third dose in generating a long-term immune response. However, this finding can also be related to the longer vaccination–delivery interval observed in this group. The low levels of antibodies for the women in Group D correlate with the waning immunity due to the extended period between the timing of the second dose and the delivery, thus showing the necessity and advantage in receiving the third dose for increased antibody levels.

Our results show a significant amount of pregestational diabetic women in Group A compared with the other groups. It is well documented that COVID-19 infection in pregnant women with comorbidities, such as pregestational diabetes, may lead to severe illness and an unfavorable outcome [24,25]. There is no solid evidence regarding higher rates of COVID-19 infection or lower antibody levels in pregestational diabetic pregnant women compared with nondiabetic pregnant women. We believe this finding in our cohort is due to the relatively small sample size of Group A (n = 10) and that this finding is not of real clinical significance.

Our results coincide with previous reports showing antibody levels among pregnant women to be significantly higher after the third dose compared to the second dose [19,20,26] and significantly higher after the second dose compared to recovered women [9,12,27]. Similar patterns were demonstrated in neonatal antibody levels [9,12,19,20,27].

Our results may imply better protection from COVID-19 to mothers and neonates due to vaccination, especially after the third dose. The effectiveness of the vaccine’s first two doses in pregnancy has been well established with a 96% reduction in the risk of any documented SARS-CoV-2 infection and a 89% reduction in the risk of severe COVID-19 disease [8]. In nonpregnant patients, the addition of a third dose of the vaccine proved effective in preventing COVID-19-related admission to hospital, severe disease, and death [17]. Published reports have confirmed the enhanced humoral response of almost all patients to the third dose, especially in populations with a low humoral response or waning immunity after the first and second doses [28,29,30]. In pregnant women and their neonates, recent studies have shown that similar to the first and second doses of the BNT162b2 vaccine, the third dose is successful in generating a remarkable humoral immune response of SARS-CoV-2 IgG, as well as being safe [18,19,20]. Moreover, a Norwegian population-based cohort study suggested there was a lower risk of a positive test for SARS-CoV-2 during the first 4 months of life among infants born to mothers who were vaccinated during pregnancy [31].

This study confirms the third dose of Pfizer/BioNTech BNT162b2 vaccine’s ability to generate immunogenicity in pregnant women and transfer antibodies to their infants. The data presented here may encourage health providers to issue recommendations regarding the vaccination of pregnant women with the vaccine’s third dose. However, large trials evaluating the clinical efficacy of the vaccine’s third dose in pregnancy are required.

This is a prospective study assessing maternal and neonatal immune responses for different recovered or vaccinated pregnant women. The strengths of this study include its prospective assessment of several different groups of pregnant women, including women vaccinated with the third dose of the vaccine. To the best of our knowledge, no such comparison has been reported to date. All serology samples were processed at the same laboratory, reducing the possibility of bias and data mismatch.

This study has several limitations: The relative short time frame and small sample size of some of the groups. The IgG tests identify the binding of WT SARS-CoV-2, which does not represent the binding of other variants. However, several studies have defined the fold decrease in neutralization of the different variants compared to WT [32,33], and a high correlation has been demonstrated between neutralization and binding [34,35,36]. The comparison of the different groups presented in this study required comparing women in different time periods. Additionally, breakthrough infections in the vaccinated groups could not be ruled out, yet we believe there was only a minor misclassification, because there was high motivation at the time to be tested, to be detected, and to receive a green pass. Furthermore, this study does not provide proof of the clinical efficacy of maternal vaccination in protecting mothers and infants from COVID-19.

## 5. Conclusions

This study compared the maternal and neonatal humoral immune responses among different groups of vaccinated, nonvaccinated, and COVID-19-recovered parturient women at the time of delivery and demonstrated that pregnant women fully vaccinated with three dosages of the BNT162b2 mRNA vaccine before delivery generated the highest levels of SARS-CoV-2 IgG antibodies and transferred the highest levels SARS-CoV-2 IgG antibodies to their infants. These data suggest an additional benefit of the third (booster) dose of the vaccine in potentially providing protection to mothers and newborns. Further research is needed to determine the optimal time for vaccination during pregnancy and to assess the clinical significance of the humoral response in reducing COVID-19-related maternal and neonatal illness and complications.

## Figures and Tables

**Table 1:** Baseline maternal and obstetric characteristics.

Characteristic	Group A “Recovered” (N = 10)	Group B “Second Vaccination” (N = 57)	Group C “Third Vaccination” (N = 38)	Group D “No Third Vaccination” (N = 8)	*p* Value
Age (years)	33.7 +/− 4.94	33.7 ± 5.85	32.2 ± 3.82	34.3 ± 3.89	0.75
BMI (kg/m^2^)	31.4 ± 5.93	28.4 ± 5.09	23.6 ± 4.31	22.4 ± 1.90	0.265
Autoimmune Disease	1 (10%)	8 (12.5%)	6 (15.8%)	2 (25%)	0.767
Lung Disease	0 (0%)	4 (6.3%)	0 (0%)	0 (0%)	0.305
Pregestational Diabetes	2 (20%)	1 (1.6%)	0 (0%)	0 (0%)	0.003 ^†^
Cardiovascular Disease	1 (10%)	2 (3.1%)	1 (2.6%)	0 (0%)	0.632
Smoking	1 (10%)	4 (6.3%)	3 (7.9%)	0 (0%)	0.839
Gestational diabetes mellitus	2 (20%)	2 (3.1%)	6 (15.8%)	0 (0%)	0.056
Hypertension disorders of pregnancy	0 (0%)	0 (0%)	3 (7.9%)	0 (0%)	0.084

Data are given as mean ± SD or n (%). BMI, body mass index. ^†^ Significance between Group A and the other groups only.

**Table 2:** Maternal and neonatal blood serology for SARS-CoV-2-specific antibodies.

	Group A “Recovered” (N = 10)	Group B “Second Vaccination” (N = 57)	Group C “Third Vaccination” (N = 38)	Group D “No Third Vaccination” (N = 8)
Maternal serum IgG (BAU/mL) ^†^	113.5 (61.62–209.1)	333.5 (327–340.2)	741.6 (514.5–1069)	57.99 (32.93–102.1)
Neonatal serum IgG (BAU/mL) ^^^	109 (49.01–242.6)	322.6 (305.6–340.5)	1269 (953.4–1690)	103.9 (48.59–222)

Data are given as geometric mean titer (GMT) and 95% confidence interval. IgG, immunoglobulin G; BAU/mL, binding antibody units per milliliter. ^†^ Significance between Group A and Group B, Group A and Group C, Group B and Group C, Group B and Group D, Group C and Group D. ^^^ Significance between Group A and Group B, Group A and Group C, Group B and Group C, and Group C and Group D.

**Table 3:** Gestational age at vaccination and vaccination–delivery time interval.

	Group A “Recovered” (N = 10)	Group B “Second Vaccination” (N = 57)	Group C “Third Vaccination” (N = 38)	Group D “No Third Vaccination” (N = 8)	*p* Value
Gestational age at vaccination/infection (weeks)	28.49 +/− 3.74	36.35 ± 1.86	23.81 ± 6.84	N/A ^†^	<0.001
Time interval between vaccination/infection to delivery (days)	118.0 ± 122.22	29.67 ± 55.99	106.95 ± 50.10	245.0 ± 64.0	<0.001 ^^^

Data are given as mean ± SD. ^^^ Significance between Group B and Group C, Group B and Group D, and Group C and Group D. ^†^ Gestational age not available for Group D as second vaccination was given before current pregnancy.

## Data Availability

The data presented in this study are available on request from the corresponding author.
